# The Evolving Role of Artificial Intelligence in Fracture Diagnosis and Surgical Planning in Orthopaedics: Current Insights and Future Directions

**DOI:** 10.7759/cureus.107470

**Published:** 2026-04-21

**Authors:** Adeolu A Badejo, Michael O Kolade, Kenechukwu Igbokwe, Timilehin Olowookere, Abiodun C Adegbesan, Imri G Adefokun, Tosin Fakiyesi, Olorungbami K Anifalaje, Funbi Ayeni

**Affiliations:** 1 Trauma and Orthopaedic Surgery, Newcastle Upon Tyne Hospitals NHS Foundation Trust, Newcastle, GBR; 2 General Surgery, Scarborough General Hospital, Scarborough, GBR; 3 Oral and Maxillofacial Surgery, South Eastern Health and Social Care, Belfast, GBR; 4 Global Health, African Cancer Institute, Cape Town, ZAF; 5 Surgery, Bowen University, Iwo, NGA; 6 Orthopaedic Surgery, Bowen University Teaching Hospital, Ogbomoso, NGA; 7 General Medicine, Shirley Oaks Circle Hospital, London, GBR; 8 Surgery, Mid Yorkshire NHS Foundation Trust, England, GBR; 9 Trauma and Orthopaedics, Imperial College Healthcare NHS Trust, London, GBR

**Keywords:** artificial intelligence, fracture detection, machine learning, pre-surgical planning, trauma and orthopaedic surgery

## Abstract

Orthopaedic fracture diagnosis and surgical planning are being revolutionised by artificial intelligence (AI) and machine learning (ML). These technologies enable the automated detection and classification of fractures and provide surgeons with AI-assisted pre-operative decision-making support at multiple body sites. The objective of this review is to synthesise current evidence (from 2016 to 2026) related to the application of AI/ML technologies in orthopaedic fracture diagnosis and surgical planning, to summarise the primary clinical findings from these studies, and to describe the challenges associated with translating these technological advancements into clinical practice.

A MEDLINE/PubMed literature search was conducted utilising a combination of Medical Subject Headings (MeSH) terms for AI, deep learning, fracture detection, and orthopaedic surgical planning. A total of 24 peer-reviewed publications that met the inclusion criteria and were published between 2016 and 2026 were identified and then synthesised through a narrative approach. Convolutional neural network (CNN)-based and Vision Transformer (ViT)-based deep learning models demonstrated area under receiver operating curves (AUROC) values ranging from 0.85 to 0.98 for detecting fractures in the hips, femurs, spines, scaphoids, tibias, pelvises, and ribs on radiographs and computed tomography (CT). The AI-assisted pre-operative planning tools have also been validated for use with pelvic and proximal humeral fractures. Additionally, ML has predicted the outcomes of surgery with AUROC values ranging from 0.78 to 0.87. There are several key limitations to the adoption of these technologies in clinical practice, including the majority of studies having been designed as retrospective analyses, the relative lack of diversity within their datasets, and the evolution of regulatory frameworks governing the application of AI/ML in medical imaging. Overall, AI technology has been shown to be highly accurate in both diagnosing fractures and assisting surgeons with planning for orthopaedic surgery. Before widespread clinical use can begin, prospective multi-centre validation and regulatory clarity will need to be achieved.

## Introduction and background

Fractures are among the most common disorders treated in orthopaedic clinics; hip fractures alone affect over 1.6 million people per year; elderly patients affected by hip fractures have a 20%-30% mortality within one year of injury [1]. Furthermore, delays in making a fracture diagnosis result in decreased treatment outcomes; it is estimated that missed fractures account for 3%-12% of radiographic interpretations; however, the majority of “occult” fractures are located in the scaphoid, ribs, and vertebrae, which pose the greatest difficulty in making an accurate diagnosis of such injuries [2]. Finally, surgical planning for complex fractures, i.e., pelvic ring injuries, intra-articular fractures, and proximal humeral fractures, requires highly sophisticated 3D anatomical reasoning skills that require significant resources and experience.

The use of artificial intelligence (AI), specifically deep learning using convolutional neural networks (CNNs), has revolutionised medical imaging [3]. In orthopaedics, AI algorithms trained on large datasets containing fractures can be used to detect fractures and classify fracture types based on AO Foundation/Orthopaedic Trauma Association (AO/OTA) criteria, and help with pre-operative segmentation of fracture fragments from computed tomography (CT) images and to assist with pre-operative surgical planning for complex fractures with accuracy similar to that of specialist surgeons [3,4]. Although there have been numerous studies demonstrating the potential of AI in orthopaedics, many clinicians remain hesitant to adopt this technology due to the lack of prospective studies, heterogeneity of training datasets, and unclear regulatory guidelines [5]. This narrative review synthesises 24 peer-reviewed publications (2016-2026) to evaluate the current state of AI in orthopaedic fracture diagnosis and surgical planning and to outline key future directions.

Methods

A literature search of PubMed/MEDLINE was conducted in March 2026 using Medical Subject Headings (MeSH) and Boolean operators. The search strategy included terms such as “artificial intelligence”, “deep learning”, “convolutional neural network”, “fracture detection”, “fracture classification”, and “orthopaedic surgical planning”. Articles published between January 2016 and March 2026 were considered. Filters were applied to include English-language, peer-reviewed articles involving human subjects. Inclusion criteria comprised English-language, peer-reviewed original research, systematic reviews, or narrative reviews addressing AI or machine learning (ML) in musculoskeletal fracture diagnosis or orthopaedic surgical planning. Exclusion criteria included non-English publications, conference abstracts, editorials, letters, case reports, studies unrelated to musculoskeletal fracture diagnosis or orthopaedic surgical planning, and studies not involving artificial intelligence or machine learning methods. Duplicate records and articles without full-text availability were also excluded. Twenty-four articles meeting these criteria were included and synthesised narratively, grouped into five thematic domains.

Because this study was conducted as a narrative review, a formal risk-of-bias tool was not applied. Instead, methodological quality was considered qualitatively by prioritising higher levels of evidence. Due to substantial heterogeneity in study design, patient populations, treatment protocols, and reported outcome measures across the included studies, quantitative pooling of data was not performed, and findings were synthesised narratively.

## Review

AI in plain radiograph-based fracture detection

Plain radiography is the most widely used and thus most well-researched imaging modality for evaluating fractures and has been shown to have high performance using AI-based computer vision techniques [6]. The ability of convolutional neural network (CNN)-based models to accurately detect fractures with sensitivities and specificities of greater than 85%-90% in comparison to other junior radiologists or even comparable to those of senior radiologists is supported by multiple systematic review articles [6,7]. A major limitation of previous studies utilising deep learning models in the field of orthopaedic imaging was determined by Sharma, who found that there was a lack of standardised benchmarking protocol across studies, making it difficult to make cross-study comparisons [8]. In addition to improving upon the accuracy of clinicians’ diagnosis of femoral fractures classified as AO/OTA classifications on plain radiograph, Tanzi et al. found that providing the clinician with the predictions of the Vision Transformer (ViT) model as decision support resulted in a 29% improvement in the clinician’s diagnostic accuracy (p = 0.003) [9].

Site-specific AI performance

For hip fractures, which are the most clinically urgent fracture, Lex et al., in a systematic review and meta-analysis of 18 studies, found that a pooled sensitivity of 0.91 and a pooled specificity of 0.93 were reported from plain radiographs; further, AI-based outcome prediction models had an area under the receiver operating curve (AUROC) values of 0.80-0.87 [1]. There are many AI-based tools to address scaphoid fractures, as they can be difficult to identify on first radiographs, with a missed detection rate of up to 20%. Yang et al. developed a two-stage convolutional neural network (CNN) where Faster R-CNN was used to detect bones and ResNet was used to classify fractures, with an AUC of 0.92 for scaphoid fracture detection [10]. Orji et al., in their systematic review, also stated that machine learning algorithms obtain sensitivities greater than 85% for the detection of scaphoid fractures and significantly outperformed emergency physician diagnoses [11]. Tibial plateau fractures have been studied by van der Gaast et al., who demonstrated deep learning could perform at least as well as experienced radiologists, particularly when it came to identifying split depression fractures [12]. Hong et al. created a VERTE-X deep learning model to use on lateral spine radiography to detect vertebral fractures and osteoporosis, demonstrating AUROCs of 0.92 and 0.93 for these conditions, respectively, while outperforming standard clinical prediction models [13].

CT-based fracture analysis and segmentation

Computed tomography (CT) is able to provide much higher levels of anatomically accurate information when compared to other imaging modalities for complex fractures; as such, AI has been used in the development of automated fragment segmentations and three-dimensional morphological characterisation of fractures from CT data [4]. Fan et al. demonstrated that deep learning-based segmentation algorithms, primarily U-Net-based architectures, can be used to automatically identify fractured bone segments from CT image volume data with Dice similarity coefficient values of ≥0.90 for delineating individual fracture fragments; these segmentation results may be used to create anatomical models for use in computer-assisted surgical planning and intra-operative navigation [4]. Kim et al. validated the ability of deep learning-based segmentation to automatically identify tibial and fibular fracture fragments on CT images, regardless of the complexity of the fracture pattern, and produced segmentation results directly applicable to pre-operative planning [14]. Dankelman et al. provided a technical framework for the use of CT fracture AI in clinical settings, emphasising the need for multi-class classification, localisation of fracture fragments at a level comparable to a single fracture fragment, and integration of fracture segmentation results with surgical navigation systems [15].

AI in pre-operative surgical planning

Pre-operative planning using AI has been shown to be viable in clinically complicated fracture types [16-18]. Liu et al. designed an all-encompassing virtual fracture reduction pipeline for pelvic ring injuries that produced anatomically valid models of bone position with clinically acceptable variability and demonstrated its potential as a feasible tool for image-guided surgical workflow [16]. Additionally, Ju et al. utilised deep learning deformable registration to automatically identify iliosacral screw corridors; their results indicated that the AI-generated results were similar to those obtained by trained orthopaedic surgeons, with a significant amount of time savings compared to manual planning methods [17]. Jeon et al. demonstrated the validation of AI-based virtual reduction of Neer three- and four-part proximal humerus fractures; they reported a positive correlation between the AI-generated plans and the quality of intra-operative reduction and post-operative results [18]. Furthermore, AI-based tools are used in spine surgery to assist in calculating trajectories for pedicle screws, identifying vertebral levels, and determining corrective angles for spinal deformities, reducing fluoroscopic radiation exposure [19].

Machine learning for outcome prediction

Beyond fracture detection, ML has also been used for predicting fracture healing, the risk of implant failure, and the rate of complications during surgery [3]. Retrospective ML models developed by Misir and Yuce to predict orthopaedic outcomes had an area under the receiver operator curve (AUROC) of 0.78-0.87; however, the amount of evidence from studies validating ML models with different population demographics is currently limited [3]. Mohabey et al. have shown that ML models can be used to predict post-operative alignment correction and patient satisfaction after high tibial osteotomy using an AUROC of 0.78-0.85, potentially allowing surgeons to plan their surgical approach based on each patient’s unique anatomy [20].

Table 1 summarises the performance metrics and clinical application of AI in orthopaedic fracture diagnosis and surgical planning.

**Table 1:** Summary of key performance metrics and clinical application of AI in orthopaedic fracture diagnosis and surgical planning AI: artificial intelligence, AUROC: area under the receiver operating characteristic curve, CNN: convolutional neural network, CT: computed tomography, DICE: Dice similarity coefficient, Sens: sensitivity, Spec: specificity, AUC: area under the curve, DXA: dual-energy X-ray absorptiometry, ED: emergency department, AO/OTA: AO Foundation/Orthopaedic Trauma Association, ML: machine learning, DL: deep learning, AR: augmented reality, ViT: Vision Transformer, MRI: magnetic resonance imaging

Author (year)	Fracture/topic	AI method	Imaging	Key performance metric	Clinical application
Lex et al. (2023) [1]	Hip	CNN ensemble	X-ray	Pooled Sens: 0.91, Spec: 0.93, outcome AUROC: 0.80-0.87	Detection and outcome prediction
Kalmet et al. (2020) [2]	Multiple sites	Deep learning (narrative review)	X-ray/CT	3%-12% miss rate addressed by AI	Fracture detection overview
Misir and Yuce (2025) [3]	Multiple/general	ML/AI (comprehensive review)	Multiple	Outcome prediction AUROC: 0.78-0.87	Comprehensive orthopaedic AI review
Fan et al. (2023) [4]	Spine, arthroplasty, fracture	AI/DL/AR/robotics	CT/X-ray	DICE: >0.90 for segmentation	Image-guided surgery and 3D planning
Glinkowski et al. (2026) [5]	Multiple	AI (systematic review)	Multiple	Clinical performance benchmarking	Translational readiness assessment
Bhatnagar et al. (2024) [6]	Multiple fracture types	CNN (review)	X-ray	Sens/Spec: >85%-90%	Fracture detection review
Lo Mastro et al. (2024) [7]	Multiple sites	CNN (literature review)	X-ray	AI equals/exceeds senior radiologist	Detection versus clinician comparison
Sharma (2023) [8]	Multiple orthopaedic	CNN/AI	X-ray	Standardised benchmarking identified as key unmet need	Current developments and future directions
Tanzi et al. (2022) [9]	Femur	ViT	X-ray	Accuracy: 83%, clinician accuracy: +29% (p = 0.003)	AO/OTA fracture classification
Yang et al. (2022) [10]	Scaphoid	Two-stage CNN (Faster R-CNN + ResNet)	X-ray	AUC: 0.92	Detection and classification
Orji et al. (2023) [11]	Scaphoid	ML algorithms (systematic review)	X-ray	Sens: >85%, exceeds ED physicians	Detection systematic review
van der Gaast et al. (2025) [12]	Tibial plateau	DL	CT	Comparable to an experienced radiologist	Detection and classification
Hong et al. (2023) [13]	Vertebral/spine	CNN (VERTE-X)	Lateral X-ray	AUROC: 0.92-0.93	Detection and DXA referral guidance
Kim et al. (2023) [14]	Tibia/fibula	U-Net deep learning	CT	DICE: >0.90	Fracture fragment segmentation
Dankelman et al. (2022) [15]	Multiple (CT fractures)	AI/CNN (review and framework)	CT	Technical requirements defined	Validation framework for CT fracture AI
Liu et al. (2025) [16]	Pelvis	Virtual reduction AI pipeline	CT	Clinically acceptable tolerances	Pre-operative fracture reduction planning
Ju et al. (2025) [17]	Pelvis (iliosacral)	Deformable registration DL	CT	Accuracy equal to experienced surgeon	Automated screw corridor planning
Jeon et al. (2024) [18]	Proximal humerus	Virtual reduction AI	CT	High intra-operative correlation	Pre-operative virtual reduction planning
Lee et al. (2024) [19]	Spine	AI/DL (review)	X-ray/CT/MRI	Review of recent advances	Spinal imaging and patient care
Mohabey et al. (2025) [20]	High tibial osteotomy	ML predictive modelling	X-ray/CT	AUROC: 0.78-0.85	Outcome prediction and surgical planning
Luo et al. (2026) [21]	Multiple	Multimodal AI	CT/X-ray/MRI	Review of technological advances	Multimodal imaging and clinical translation
Rohde and Münnich (2022) [22]	Multiple	AI (review)	X-ray/CT	Narrative overview of AI performance	Orthopaedic and trauma imaging review
Vaishya et al. (2025) [23]	Multiple	AI integration (review)	Multiple	Opportunities and challenges reviewed	Clinical AI integration in orthopaedics
Akram et al. (2025) [24]	Spine	Computer vision (scoping review)	X-ray/CT/MRI	Scoping review of imaging algorithms	Diagnosis, measurement, and surgical planning

Discussion

This review demonstrates that AI has provided clinical utility across three related areas of orthopaedic practice: fracture detection, fracture classification, and pre-operative surgical planning [3-5]. AI has shown high levels of sensitivity and specificity in detecting fractures (approaching or equalling those of expert radiologists) across multiple anatomical regions (hip, scaphoid, spine, tibia, pelvis, and ribs) [6,7]. AI can classify fractures into existing taxonomies (such as the AO/OTA classification) at levels of accuracy equivalent to trained orthopaedic surgeons and thus reduce the long-standing interobserver variability that has impacted the ability to make consistent clinical decisions [9]. AI also enables the rapid development of virtual fracture reduction pipelines and automated implant corridors for the identification of appropriate fixation options for pelvic ring injuries, proximal humeral fractures, and spinal instrumentation with clinically acceptable accuracy [16-18]. Together, these advances indicate a significant advancement in the clinical capability of AI within orthopaedic trauma care [3,5].

In almost every case reviewed, the use of AI as a decision support tool when used as part of a human-AI collaboration resulted in greater diagnostic accuracy than the use of AI alone or the use of human expertise alone [9,18]. This finding is consistent across fracture type, anatomical site, and algorithm architecture, and represents one of the most clinically relevant findings of this body of literature. For example, Tanzi et al. found that orthopaedic clinicians who were supported by Vision Transformer predictions improved their AO/OTA femur fracture classification accuracy by 29% (p = 0.003) compared to unaided assessment and had a combined human-AI accuracy of 97%, significantly improving upon the algorithm alone (which was 83%) [9]. Similarly, Jeon et al. found that surgeons who utilised AI-generated virtual reduction plans for Neer three- and four-part proximal humeral fractures achieved statistically similar reductions of the fracture during surgery as predicted by the AI (with strong post-operative radiographic correlation) [18]. As these studies illustrate, the idea of “augmented intelligence”, where AI enhances the accuracy, consistency, and reproducibility of clinical judgment, rather than replacing it, is being realised [5]. Therefore, the practical implication is that AI tools should be developed and implemented as interactive decision support systems integrated into clinical workflows, rather than as standalone diagnostic engines [3,5].

In addition, future generations of AI will involve multimodal AI architectures that combine radiologic images with clinical variables, patient self-reported measures, and functional data, representing the next level of orthopaedic AI capability, and impacting both diagnosis and prognosis [21]. Current AI tools primarily analyse plain radiographs or CT images in isolation and therefore do not capture the complete clinical picture that guides orthopaedic decision-making [4,8]. New multimodal architectures reviewed by Luo et al. combine radiographic, MRI, and structured clinical data into a single deep learning framework and have shown early superiority over unimodal imaging AI for fracture risk stratification and surgical outcome prediction [21]. It is anticipated that new multimodal AI capabilities will increase the clinical utility of AI and move the technology from a single-task diagnostic aid to a comprehensive clinical intelligence platform that supports the entire patient pathway [21].

The biggest barriers to translating AI research into clinical use include the lack of representation of the populations in which the algorithms have been trained [5]. Most algorithms have been trained using retrospective single-centre imaging datasets [22]. These single-centre datasets are often created by hospitals and universities located in wealthier countries, with access to standardised imaging equipment, and primarily include young and healthy individuals [22]. Therefore, it is unlikely that these models can be effectively translated to other imaging modalities, other patient populations (such as elderly patients with osteoporosis, paediatric patients with open physis, or patients who have had previous orthopaedic implants), or different clinical settings utilising older or lower specification imaging equipment [2,5]. Furthermore, when testing these algorithms on external validation groups obtained from different centres, performance consistently decreases due to what has come to be known as “domain shift”. Domain shift occurs when statistical relationships are learned in one data distribution during training and then fail to generalise to other unseen data distributions [15]. Dankelman et al. advocated for the creation of multi-institutional prospective datasets and standardised validation protocols before any AI system for clinical-grade CT fracture detection is deemed ready for clinical use; most current tools have not met these standards [15]. Achieving this goal requires cooperative funding for the development of prospective multi-centre imaging databases and the establishment of free, widely available, and thoroughly annotated orthopaedic imaging databases for use by the global research community [21,23].

Another significant impediment to translating AI technology into clinical use is a lack of transparency in how the algorithms work. Transparency refers to the ability of clinicians and regulatory reviewers to understand how a model generates its predictions and to verify those predictions against established clinical standards [22]. While contemporary deep learning architectures demonstrate excellent predictive capability, they also represent “black boxes” that do not inherently generate explanations for their predictions in a form that clinicians can understand, question, or alter [8]. Tools such as gradient-weighted class activation mapping (Grad-CAM) offer some degree of visualisation of the image areas that contribute to model predictions [7,22]. However, Grad-CAM has not been compared to clinical decision-making in orthopaedics [7,22]. Without sufficient transparency, clinicians cannot fulfill their obligation to assume accountability for decisions made with the assistance of AI, nor can regulatory bodies validate the safety and effectiveness of these tools for use without direct supervision in clinical settings [5,22]. Therefore, the development of standardisation for explanation frameworks must be considered an indispensable prerequisite for wide-scale adoption [5,22].

Finally, the regulatory pathway for the approval of AI-based SaMDs is changing rapidly, but remains disparate and unclear across various jurisdictions globally, and creates ongoing uncertainty for both AI developers and healthcare organisations interested in implementing AI tools [23,24]. For example, the FDA’s Predetermined Change Control Plan provides a conceptual framework for accommodating the dynamic, adaptive nature of machine learning algorithms and represents a significant departure from the static approval process traditionally used to evaluate medical devices [23]. However, the European Union’s AI Act establishes a risk-stratified regulatory framework for AI tools intended for use in making clinical decisions; however, there is a dearth of detail provided in the literature with regard to the specifics of the application of this framework to orthopaedic AI applications [23]. Post-market surveillance criteria for AI systems that update their parameters as additional clinical data becomes available (“locked” versus “adaptive”) have not been clearly articulated in any jurisdiction [5,23]. Vaishya et al. have proposed that regulatory frameworks for AI in medicine should be developed via a collaborative approach with multidisciplinary participation (clinicians, engineers, patients, ethicists, etc.) in the design of AI tools from the outset of development, rather than developing them in isolation after the fact [23]. However, until regulatory frameworks are clarified and post-market surveillance criteria are defined, developers and organisations will continue to experience uncertainty with respect to compliance issues and thus delay the implementation of tools that have demonstrated benefits [5,23,24].

## Conclusions

AI has the potential to have a very positive impact on orthopaedics as it can help reduce the number of missed fractures, improve how we classify fractures, and provide better pre-operative surgical planning for orthopaedic patients. The translation of these benefits into clinical practice will require well-designed prospective, multi-centre validation studies and development of training datasets that are representative of diverse and inclusive populations. Additionally, regulatory frameworks need to be developed to support the deployment of AI throughout orthopaedic care. These regulatory efforts should include collaboration with multiple stakeholders (i.e., orthopaedic surgeons, radiologists, biomedical engineers, etc.) so that AI is implemented safely, fairly, and effectively at all levels of orthopaedic care.
